# Cofactor-mediated amyloidogenesis

**DOI:** 10.1042/BSR20190327

**Published:** 2019-03-06

**Authors:** Jillian Madine

**Affiliations:** Institute of Integrative Biology, University of Liverpool, Liverpool, U.K.

**Keywords:** amyloid, glycosaminoglycans, metal ions, membrane-interactions, protein aggregation

## Abstract

A recent study published in Bioscience Reports by Sheng et al. (Bioscience Reports, (2019) **39**, pii:BSR20182345] described a small but significant conformational change that occurs upon zinc binding and results in initiation of the amyloidogenic aggregation cascade of Golgi-Associated plant Pathogenesis Related protein 1 (GAPR-1) in the presence of heparin. The present study describes a two-stage process that is required for the initiation of the amyloidogenic aggregation cascade involving a concentration step and a conformation change to enhance accessibility of natively protected amyloidogenic regions for self-association. For GAPR-1 in the present study, these steps are provided by zinc binding causing the required conformational change enhancing accessibility of amyloidogenic regions, and heparin providing a template or scaffold in turn increasing the local protein concentration. Cofactors such as glycosaminoglycans and metal ions have been found associated with amyloid deposits *in vivo* and shown to affect protein assembly kinetics *in vitro.* Cofactor interactions with the amyloidogenic process are an area of great interest for therapeutic intervention for the wide range of diseases known to be associated with amyloid protein aggregation. The present study emphasises the need for enhanced structural understanding of cofactor–amyloid protein interactions and highlights that small subtle conformational changes can have large impacts on resulting aggregation processes.

Golgi-Associated plant Pathogenesis Related protein 1 (GAPR-1) is a member of the CAP (cysteine-rich secretory proteins, Antigen 5, and Pathogenesis-related 1 protein) family, in which several members have been described to form amyloid-like structures in the presence of lipids. In addition to having amyloidogenic properties itself GAPR-1 has also been shown to inhibit aggregation of Aβ(1-40) peptide by binding to prefibrillar oligomeric Aβ structures during the early stages of fibril formation [1]. It is thought that this may be due to natively folded GAPR-1 possessing an intrinsic amyloid-related structure identified through binding to the amyloid oligomer-specific antibody A11 [1], thus enabling association between the oligomeric Aβ and native GAPR-1. Authors of the recent report also test additional CRISP subfamily proteins possessing a CAP domain and show that in two out of three, a similar increase in amyloidogenic aggregation in the presence of zinc and heparin was observed. It is therefore hypothesised that the CAP domain may regulate protein oligomerisation in a large variety of proteins that define the CAP superfamily.

Amyloid fibrils and deposits, made up of over 30 proteins and peptides, are the pathological hallmark of many human disorders [2]. The precise nature of the pathogenic amyloid species and the role of amyloidogenic aggregation in disease initiation, progression and cytotoxicity is a matter of intense debate and the focus of many research groups. It is well accepted that cofactors play a role in protein assembly and amyloidogenic aggregation, and that these cofactors may represent targets for therapeutic intervention for the wide range of diseases known to be associated with amyloid protein aggregation. Three key factors that are well established to influence amyloidogenic aggregation *in vitro* and have been found associated with deposits *in vivo* are metal ions, membranes and glycosaminoglycans.

Metal ions (Cu^II^, Zn^II^, Fe^III^) are found associated with amyloid deposits, e.g. in Alzheimer’s disease brains [3] with direct binding to amyloid proteins (e.g. Aβ) and association with fibrils reported. Metal ions contribute to amyloid formation and associated disease pathology via two distinct mechanisms: alteration of amyloid assembly and direct effect on enhancement of cytotoxicity. Metal ions may influence amyloid assembly in several different ways: (i) Metal-induced conformational change. (ii) Metal-induced cross-linking. (iii) Metal-induced neutral net charge. (iv) Metal-induced change in fibre morphology. (v) Metal-induced change in protein stability [4]. Evidence is available for all these effects in different protein systems that can be manipulated by different metal ions, varying concentrations, and surrounding conditions. This variation in effect could be linked to how metal ions actually interact with a protein, e.g. Cu^2+^ ions accelerate fibril formation of Aβ whereas Zn^2+^ ions inhibit fibril formation [5]. Metal ions have the ability to catalyse production of H_2_O_2_ suggesting that toxicity of amyloid proteins and specifically oligomers could result from metal ion-induced H_2_O_2_ generation [6]. Metal chelators have been shown to be effective in reducing H_2_O_2_ production and amyloid accumulation *in vitro* and in animal models and pose a promising therapeutic avenue [7].

The present study of GAPR-1 in Bioscience Reports is an example of a small conformational change initiated by zinc binding that results in enhanced exposure of amyloidogenic regions in the C-terminus (distant to the zinc-binding site) and in turn self-association [8]. Metal ions are known to bind to charged residues with co-ordination often occurring via histidine residues as identified for GAPR-1. It has been shown that for proteins with acid pI’s (Aβ, α-synuclein, β2-microglobulin) amyloid fibril formation is accelerated while for basic proteins (human prion protein, islet amyloid polypeptide) fibril formation is inhibited [4]. Binding of metal ions to a protein with an acidic pI may result in net neutral charge and resultant enhanced ability to self-associate. However, in addition to promoting formation of fibrils metal ions have also been shown to induce amorphous precipitation [9]. It, therefore, appears that this pathway is under tight regulation and exists as a balance that may result in protein precipitation rather than fibril formation depending on ion concentration and degree of neutralisation.

In the recent study, GAPR-1 aggregation was induced by addition of zinc and the presence of heparin [8]; however previous work has shown that association with acidic phospholipid membranes can have the same aggregation inducing effect [1]. The recent study proposes a two-stage pathway required for initiation of amyloidogenic aggregation involving a concentration step (could be achieved via membrane association or presence of heparin to generate a template or ‘seed’) and a conformation change to enhance accessibility of natively protected amyloidogenic regions for self-association (could be induced by multiple methods, e.g. association with lipids or metal ions). Membranes and extracellular matrix components can play a critical role affecting both aspects of the aggregation initiation pathway by providing a structural template for recruitment of monomers to restrained monomers/dimers on a surface initiating self-assembly and by inducing significant conformational changes in monomers or dimers leading to the exposure of amyloidogenic segments. Charge interactions are thought to play a role in the initial step of recruitment at membrane or other, e.g. glycosaminoglycan surfaces resulting in restricted movement and increased local concentration. It is well known that Aβ membrane binding is enhanced by the presence of negatively charged lipid headgroups and that Aβ interacts with negatively charged surfaces of glycosaminoglycans [10].

The two-stage aggregation initiation hypothesis is likely to exist for all amyloid proteins with different factors initiating the steps in different pathological situations. This could explain how the same protein can assemble in different ways to form vastly different disease pathologies dependent on the initiating cofactors. Both aspects of the pathway represent avenues for future therapeutic exploration. What still needs to be determined is whether both the ‘templating’ and conformation change needs to be targeted together or whether one step is sufficient to prevent self-association in turn delaying or preventing associated pathological consequences.

The present study emphasises the need for enhanced structural understanding and that small subtle conformational changes can have large impacts on resulting aggregation processes [8]. It should be noted that GAPR-1 possesses a native intrinsic amyloid-related structure as recognised by oligomeric conformational antibody A11 [1] which may make it more susceptible to small conformational changes inducing aggregation. In contrast, when the native structure does not possess any amyloid-like properties as is the case for many known amyloid proteins, a greater conformational change is required to initiate the amyloidogenic aggregation pathway. Irrelevant as to the magnitude of conformational change required the present study highlights the need for multiple techniques to identify relevant changes and how computational studies can assist this understanding and complement experimental approaches. Understanding the impact of induced conformational changes on the amyloidogenic aggregation pathway is an essential step towards targeting initiating interactions with cofactors for therapeutic purposes.

## Abbreviations

CAP: cysteine-rich secretory proteins, Antigen 5, and Pathogenesis-related 1 protein
GAPR-1: Golgi-Associated plant Pathogenesis Related protein 1

## References

[1] Olrichs N.K., Mahalka A.K., Kaloyanova D., Kinnunen P.K. and Bernd Helms J. (2014) Golgi-Associated plant Pathogenesis Related protein 1 (GAPR-1) forms amyloid-like fibrils by interaction with acidic phospholipids and inhibits Aβ aggregation. Amyloid 21, 88–96 10.3109/13506129.2014.882304 24471790

[2] Benson M.D., Buxbaum J.N., Eisenberg D.S., Merlini G., Saraiva M.J.M., Sekijima Y. (2019) Amyloid nomenclature 2018: recommendations by the International Society of Amyloidosis (ISA) nomenclature committee. Amyloid 25, 215–219 10.1080/13506129.2018.1549825 30614283

[3] Lovell M.A., Robertson J.D., Teesdale W.J., Campbell J.L. and Markesbery W.R. (1998) Copper, iron and zinc in Alzheimer’s disease senile plaques. J. Neurol. Sci. 158, 47–52 10.1016/S0022-510X(98)00092-6 9667777

[4] Viles J.H. (2012) Metal ions and amyloid fiber formation in neurodegenerative diseases. Copper, zinc and iron in Alzheimer’s, Parkinson’s and prion diseases. Coordin. Chem. Rev. 256, 2271–2284 10.1016/j.ccr.2012.05.003

[5] Sarell C.J., Wilkinson S.R. and Viles J.H. (2010) Substoichiometric levels of Cu^2+^ ions accelerate the kinetics of fiber formation and promote cell toxicity of amyloid-β from Alzheimer disease. J. Biol. Chem. 285, 41533–41540 10.1074/jbc.M110.171355 20974842 PMC3009880

[6] Opazo C., Huang X., Cherny R.A., Moir R.D., Roher A.E., White A.R. (2002) Metalloenzyme-like activity of Alzheimer’s disease β-amyloid: Cu-dependent catalytic conversion of dopamine, cholesterol, and biological reducing agents to neurotoxic H_2_O_2_. J. Biol. Chem. 277, 40302–40308 10.1074/jbc.M206428200 12192006

[7] Lee J.Y., Friedman J.E., Angel I., Kozak A. and Koh J.Y. (2004) The lipophilic metal chelator DP-109 reduces amyloid pathology in brains of human beta-amyloid precursor protein transgenic mice. Neurobiol. Aging 25, 1315–1321 10.1016/j.neurobiolaging.2004.01.005 15465629

[8] Sheng J., Olrichs N.K., Geerts W.J., Li X., Rehman A.U., Gadella B.M. (2019) Zinc binding regulates amyloid-like aggregation of GAPR-1. Biosci. Rep. 39, pii: BSR20182345, 10.1042/BSR20182345 PMC6900432 30700571

[9] Pedersen J.T., Østergaard J., Rozlosnik N., Gammelgaard B. and Heegaard N.H.H. (2011) Cu(II) mediates kinetically distinct, non-amyloidogenic aggregation of amyloid-β peptides. J. Biol. Chem. 286, 26952–26963 10.1074/jbc.M111.220863 21642429 PMC3143654

[10] Madine J., Pandya M.J., Hicks M.R., Rodger A., Yates E.A., Radford S.E. (2012) Site-specific identification of an Aβ fibril–heparin interaction site by using solid-state NMR spectroscopy. Angew. Chem. Int. Ed. (Engl.) 51, 13140–13143 10.1002/anie.201204459 23161730 PMC3749465

